# Effect of Occlusal Splint Guidance on Masseter Muscle Activity During Sleep in Adults with Sleep Bruxism: A Preliminary Randomized Crossover Clinical Trial

**DOI:** 10.3390/jcm14248799

**Published:** 2025-12-12

**Authors:** Megumi Matsuyama, Masayuki Takaba, Yuka Abe, Kohei Maejima, Shiori Okuhara, Toshiro Hirai, Kazuyoshi Baba

**Affiliations:** Department of Prosthodontics, Graduate School of Dentistry, Showa Medical University, Tokyo 145-8515, Japan

**Keywords:** dental occlusion, electromyography, masticatory muscles, occlusal splints, sleep bruxism

## Abstract

**Background/Objectives**: Occlusal splints are widely used for managing sleep bruxism (SB), providing uniform contact across the entire dentition in the centric relation. Nonetheless, different guidance schemes, such as bilateral balanced occlusion (BBO) and canine guidance (CG), are used during eccentric movements, and the optimal design remains unclear. This study compared the effects of BBO and CG on masticatory muscle activity, sleep architecture, and subjective outcomes during sleep. **Methods**: This non-blinded randomized crossover trial enrolled 24 healthy adults diagnosed with SB (16 men and 8 women; mean age, 26.1 years) who were randomly assigned to either a BBO-first or CG-first sequence. Individual splints of both types were milled from the polymethyl methacrylate discs. After a 5-night baseline period, each splint was worn for 33 nights in a home environment, and data from nights 29 to 33 were analyzed. Masseter muscle activity was assessed using single-channel electromyography (EMG), yielding EMG parameters, including integrated EMG per hour, number of episodes and bursts per hour, mean episode duration, and total episode duration per hour. Sleep architecture was assessed using portable polysomnography with automatic scoring, and subjective outcomes were assessed for sleep disturbance, morning symptoms, and splint comfort. Differences between splints were analyzed using Wilcoxon signed-rank tests (α = 0.05). **Results**: Twenty-three participants completed the study. No statistically significant differences were found between the BBO and CG splints for any EMG parameters, sleep variables, or subjective measures. **Conclusions**: Splint guidance design differences showed no significant effects; however, smaller, potentially clinically relevant effects cannot be excluded.

## 1. Introduction

Sleep bruxism (SB) is defined as masticatory muscle activity during sleep and is characterized as rhythmic (phasic) or non-rhythmic (tonic) [1]. Notably, it can cause dental problems, including tooth wear, damage to prosthetic restorations, tooth fracture, and temporomandibular disorders (TMDs) [2,3], which may compromise the long-term prognosis following prosthodontic treatments [4], thereby highlighting the importance of effective SB management in dental practice. Nevertheless, the underlying mechanisms are not fully understood, and no definitive therapeutic or preventive strategies have been established.

To date, several approaches have been proposed for SB management [2,5,6], including occlusal splint therapy [7,8,9,10,11], pharmacological treatment [12,13], biofeedback [14,15], and cognitive behavioral therapy [16]. Occlusal splint therapy is the most commonly used in clinical practice and is considered the standard conservative approach for SB management. Its use leads to a short-term reduction in masticatory muscle activity associated with SB; however, this effect is transient, and muscle activity returns to baseline levels after approximately 2 weeks of continuous use owing to adaptation [9,10]. Thus, splints are not considered effective for long-term SB suppression [11]. Nevertheless, they continue to be routinely used because they protect teeth and prosthetic restorations from the SB-generated excessive forces.

Occlusal splints are designed to provide multiple simultaneous contacts in the centric relation position, similar to stabilization splints used in TMD management. This allows for the rational redistribution of occlusal loads across the dentition, thereby protecting teeth and prosthetic restorations from excessive wear and mechanical stress [7,8,11].

Several tooth-contact schemes for eccentric mandibular movements can be incorporated into occlusal splints, including bilateral balanced occlusion (BBO), canine guidance (CG), and group function. Studies involving individuals with natural dentition have reported that CG can significantly reduce masticatory muscle activity compared with other schemes [17,18,19,20,21]. Therefore, several researchers have recommended incorporating CG into splint design [22,23]. Although these studies evaluated the effects of different occlusal guidance schemes on masticatory electromyography (EMG) activity, they were all conducted under simulated jaw movement tasks while awake [22,23,24] and did not assess actual SB-related muscle activity. Rugh et al. [25] conducted a crossover study in eight individuals with SB who wore splints with CG and molar guidance (contact limited to the first molars) for 10–14 consecutive nights. The study found no significant differences in masticatory muscle activity during sleep between the two guidance types. Nevertheless, the small sample size limits the statistical generalizability of the findings. In addition, the comparison was based on data collected immediately after splint insertion without accounting for adaptation to occlusal splints.

Current evidence indicates that SB is primarily a centrally mediated sleep-related phenomenon, driven by micro-arousals, autonomic activation, and neurotransmitter regulation rather than static occlusal characteristics [26,27,28]. Nonetheless, several reviews and observational studies have suggested that peripheral factors may modulate the expression of SB by influencing pattern shaping, load distribution, and symptom manifestation, even if they do not act as primary etiological drivers [29,30].

Classical occlusion-based theories have largely been refuted, as experimental and epidemiologic studies do not demonstrate a robust causal relationship between malocclusion, occlusal interference, and SB events. However, specific occlusal or temporomandibular characteristics, such as joint sounds or tooth inclination, have been associated with SB in recent polysomnography (PSG)-confirmed case–control studies, supporting a model of conditional peripheral modulation rather than causation [31,32].

Management-focused systematic reviews further indicate that certain oral appliances and biofeedback devices can transiently reduce EMG activity or mechanical loading [2,8,26]. These effects are interpreted as peripheral mechanical or sensorimotor modulation and interruption of centrally generated activity, rather than suppression of the central propensity to produce SB. In contrast, pharmacologic studies show that centrally acting agents can reduce SB indices [12,13], reinforcing the primacy of central mechanisms.

Taken together, the literature supports a hierarchical model in which SB originates from central pattern generators and autonomic–cortical arousal mechanisms, while peripheral factors—occlusal morphology, temporomandibular structures, oral appliances, and feedback pathways [14,15]—modulate the magnitude, direction, and clinical consequences of the motor output. Thus, although current evidence does not support a dominant peripheral role in its pathogenesis, occlusal guidance may influence the expression of SB to a limited extent.

Among the commonly used occlusal splint designs for SB, BBO and CG represent opposite extremes in lateral occlusal contact distribution: BBO provides the greatest occlusal contact area during lateral movements, whereas CG provides the smallest. In assessing the effects of guidance design, these two schemes were selected because the mechanical contrast between them is expected to be most pronounced, allowing a clear evaluation of how guidance design influences masticatory muscle activity.

This randomized crossover study aimed to compare the effects of BBO and CG splints on masticatory EMG activity, sleep architecture, and subjective outcome measures in adults with SB. The null hypothesis was that there would be no significant difference in the primary outcome, integrated EMG per hour during sleep, between splints with BBO and those with CG.

## 2. Materials and Methods

### 2.1. Ethics Statements

The study protocol adhered to the principles of the Declaration of Helsinki, the Standard Protocol Items: Recommendations for Interventional Trials (SPIRIT) Statement 2013, and the Consolidated Standards of Reporting Trials (CONSORT) Statement 2010 and was approved by Showa Medical University Research Ethics Review Board (approval number: 2024-179-A). All the participants received a full explanation of the study and provided written informed consent. The study protocol was registered with the Japan Registry of Clinical Trials (https://jrct.mhlw.go.jp/ (accessed on 9 December 2025)); trial registration number: jRCT1032240493; 18 November 2024).

### 2.2. Participants

Participants were enrolled between November 2024 and May 2025. The inclusion criteria were as follows: (1) age between 18 and 40 years; (2) students or dentists affiliated with the dental hospital of Showa Medical University; (3) presence of at least one of clinical signs and symptoms of SB, including morning masticatory muscle fatigue or pain, tooth wear, or reports of grinding sounds during sleep by a sleep partner; and (4) SB episodes ≥ 7.6 per hour, as determined by masseter EMG recording [33]. The exclusion criteria were as follows: (1) two or more missing molars, except for the third molars; (2) removable denture use; (3) ongoing medical or dental treatment, including orthodontic therapy; (4) medication use that may affect sleep; (5) alcohol or drug abuse; (6) major psychiatric or neurological disorders; (7) sleep disorders; (8) difficulty conducting in-home EMG and PSG recordings; and (9) methacrylate polymer hypersensitivity.

To assess eligibility, SB-related EMG activity was recorded at home for 3 consecutive nights using a single-channel EMG device (EMG Logger; GC Corp., Tokyo, Japan). To minimize first-night effects associated with unfamiliarity with the device [34], data from the first night were excluded. SB was diagnosed based on the presence of ≥7.6 SB episodes per hour, as determined from either the second or third night of recording [33].

### 2.3. Sample Size Calculation

Based on a previous study [24], referring to integrated EMG per hour as the primary outcome in this study, the effect size for masseter EMG activity differences during eccentric movements between group function and CG splints under awake conditions was estimated to range between 0.5 and 1.1; here, the effect size comparing EMG activity during sleep between BBO and CG splints was set at 0.8. With a type I error (α) of 0.05 and type II error (β) of 0.1, the required sample size was 19 (G*Power 3.1.9.6, Universität Düsseldorf, Germany), and the target enrollment was set at 24 to account for potential exclusions.

### 2.4. Splint Fabrication

**Figure 1:**
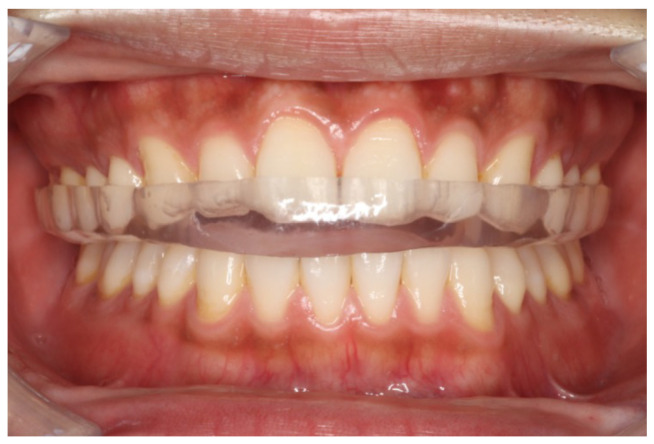
Frontal view of the splint. Occlusal splints are designed to provide multiple simultaneous contacts in the centric relation position, with a molar region thickness of 1.0–1.5 mm.

Maxillary and mandibular impressions, along with jaw relation records, were obtained for each participant. After scanning the casts fabricated from hard dental stones, maxillary occlusal splints with BBO and CG were designed using CAD software (exocad 3.2 Elefsina; exocad GmbH, Darmstadt, Germany) and milled from polymethyl methacrylate discs (Pure PMMA Disc; Quest Corporation, Tokushima, Japan). Splint thickness in the molar region was set at 1.0–1.5 mm. The splint is shown in situ in Figure 1 to visualize its intraoral fit.

**Figure 2:**
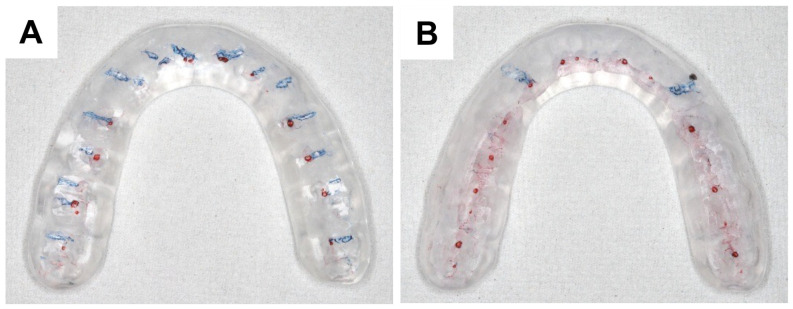
Two types of occlusal splints with different guidance designs. (**A**) Splint with bilateral balanced occlusion guidance; (**B**) Splint with canine guidance. Red marks indicate contact points in centric relation, and blue lines indicate guidance during eccentric movements.

All adjustments for BBO and CG splints were performed by a single experienced prosthodontist (M.M.). Occlusal splints were adjusted to provide multiple simultaneous contacts in the centric relation position. Lateral mandibular movements were adjusted as illustrated in Figure 2. During protrusive mandibular movements, contacts were intentionally limited to the anterior teeth, from the right to left canines, and were balanced as evenly as possible. This adjustment was applied to both CG and BBO splints, as achieving full-arch contact in the forward direction was not feasible with the BBO splint on the natural dentition.

### 2.5. Study Protocol

**Figure 3:**
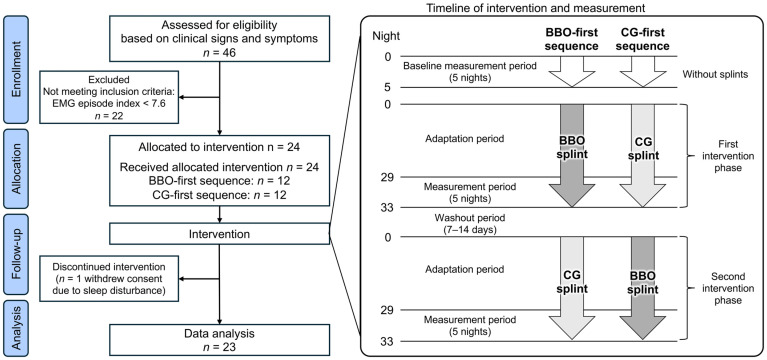
CONSORT 2010 flow diagram of participant inclusion and study interventions. BBO, bilateral balanced occlusion; CG, canine guidance; CONSORT, the Consolidated Standards of Reporting Trials.

This study employed a non-blinded randomized crossover design (Figure 3). The participants were randomly allocated in a 1:1 ratio to either the BBO-first or CG-first sequence, with sex as the stratification factor. A single dentist (M.M.) was responsible for participant selection and enrollment, and entered the required information into a web-based automated allocation system (Mujinwari; https://mujinwari.biz/ (accessed on 9 December 2025), Iruka System Co., Ltd., Tokyo, Japan). The system then automatically generated the randomization sequence and assigned participants to their respective intervention groups, ensuring allocation concealment. Due to the distinct differences in occlusal design between the two splints, blinding of both participants and clinicians was not feasible.

The study timeline consisted of an initial 5-night baseline measurement period without a splint, followed by two intervention phases. According to the randomization order, the participants wore both BBO and CG splints for 33 consecutive nights. The last 5 nights of each phase (nights 29–33) were designated as the measurement period, during which masseter EMG and electroencephalographic (EEG) signals were recorded at the participants’ homes using simultaneous EMG and portable PSG devices. To account for the substantial night-to-night variability inherent in SB [35], measurements were conducted over five consecutive nights rather than a single night, thereby minimizing the influence of daily fluctuations on the outcome measures.

A washout period of 7–14 days was implemented between the two intervention phases. In crossover trials on SB, washout periods of approximately one week have commonly been adopted for centrally acting pharmacological interventions [12]. Recent data on night-to-night variability [35] and first-night effects [34] indicate that bruxism indices fluctuate over several nights rather than weeks, and that no progressive accumulation of bruxism activity occurs over longer periods. Previous splint studies have also reported that the frequency of SB episodes typically returns to baseline within 1–2 weeks after discontinuing splint use [10], and a washout period of 1–2 weeks was adopted in a crossover study comparing Michigan and nociceptive trigeminal inhibitory splints during sleep [36]. Overall, the 7- to 14-day washout period in our study is comparable to that used in previous research.

To monitor compliance with splint use, each participant was provided with a daily record sheet prior to the study period. Participants were instructed to indicate whether the splint was worn each day and to record the duration of device use in the morning and evening, as well as any periods of nighttime awakening. A free-text section was also included for participants to provide additional comments regarding their use of the device.

### 2.6. Outcomes and Evaluation Measures

#### 2.6.1. Primary Outcome

The primary outcome was the integrated EMG per hour of the masseter muscle during sleep, measured using masseter EMG recordings.

#### 2.6.2. Secondary Outcomes

Secondary outcomes included additional EMG parameters of the masseter muscle, namely the number of episodes per hour, number of bursts per hour, mean episode duration, and total episode duration per hour. Sleep variables were also assessed using portable PSG and included total sleep time, sleep efficiency, sleep latency, wake after sleep onset, proportion of time spent in each sleep stage (rapid eye movement [REM], N1, N2, and N3), awakening index, and microarousal index. Subjective outcomes were evaluated for sleep quality and disturbances using the Japanese version of the Pittsburgh Sleep Quality Index (PSQI-J), as well as participant-reported morning symptom severity and splint comfort.

### 2.7. Masseter EMG Recordings and EMG Parameters

**Figure 4:**
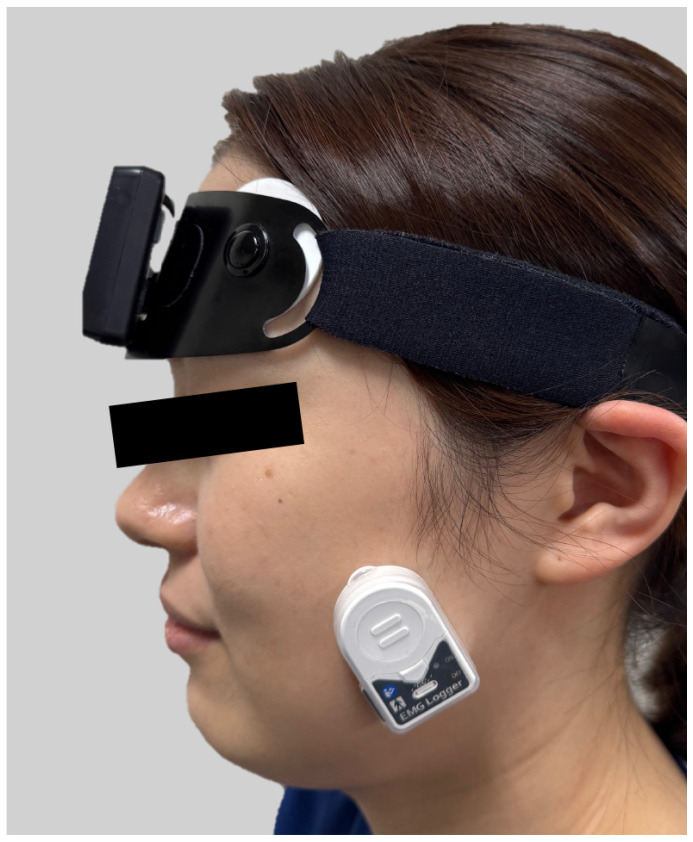
Installation of recording equipment. Participants were instructed to wear the single-channel wearable electromyography (EMG) device and portable polysomnography (PSG) system simultaneously during the measurement period.

Masseter EMG activity was recorded using the same single-channel wearable EMG device used for the diagnosis. The device had a sampling rate of 1 kHz, a high-pass filter set at 20 Hz, and 12-bit resolution [33,37]. A dentist carefully instructed the participants on how to use and attach the device to the masseter region (Figure 4). Participants were allowed to freely choose the attachment side (right or left), which was kept consistent throughout the measurement period, taking into account factors such as symptom predominance, sleep posture, electrode stability, and overall comfort. Participants were instructed to perform a maximal voluntary contraction (MVC) of the jaw muscles immediately at the onset of recording. EMG data were analyzed using W-EMG Viewer software (version 2.0.5; GC Corp., Tokyo, Japan). The first 30 min after device activation and the last 15 min before deactivation were excluded from the analysis. After selecting EMG bursts corresponding to MVC, a 3 s segment was used to determine the baseline amplitude from the EMG waveforms. SB episodes were then automatically identified, and EMG measurements were automatically calculated through software analysis. Among the automatically extracted waveforms, those with a peak amplitude at least twice the baseline but less than 300% MVC, and a duration between 0.25 and 60 s, were identified as bruxism bursts. Phasic episodes (≥3 bruxism bursts, each lasting 0.25–2.0 s), tonic episodes (a bruxism burst lasting > 2.0 s), and mixed episodes (both types of bruxism bursts) were regarded as SB episodes. Obvious artifacts or abnormal waveforms were automatically excluded by the built-in algorithm of the analysis software. The scoring results of the SB episodes were verified by visually inspecting the EMG waveforms. A single analyst (S.O.) performed these evaluations and was blinded to both the splint type and participants to prevent detection bias. Intra-rater reliability for the primary outcome, integrated EMG per hour, was high, with an intraclass correlation coefficient (ICC) of 0.947 (95% confidence interval: 0.813–0.986), confirming the consistency and reproducibility of the manual verification procedure.

Based on the self-reported sleep onset, wake-up, and nocturnal awakening times of participants, five EMG parameters were calculated: (1) integrated EMG per hour, (2) number of episodes per hour, (3) number of bursts per hour, (4) mean episode duration, and (5) total episode duration per hour.

### 2.8. Home PSG Recordings and Sleep Variables

Sleep architecture was assessed using a portable PSG device (Sleep Profiler™; Advanced Brain Monitoring, Inc., Carlsbad, CA, USA) (Figure 4) [38,39,40]. The device has been reported to achieve a mean overall agreement of 71.3% between its automated sleep staging and manual PSG scoring by five independent, registered PSG technologists [38]. Furthermore, compared with manual PSG scoring, which had a mean kappa score of 0.70 across 10 comparisons by the same five technologists, the automated scoring has been reported to yield a mean kappa score of 0.63 (range, 0.62–0.65) [38]. This battery-powered, head-mounted recorder (71 × 20 × 48 mm; 72 g) enables the simultaneous recording of multiple signals, including three frontopolar EEG signals: AF7–AF8, AF7–Fpz, and AF8–Fpz. Sleep staging was automatically performed in 30 s epochs using dedicated analysis software, and the values for each sleep variable were automatically calculated; no manual scoring or verification was performed in the present study. Obvious artifacts or abnormal waveforms were automatically excluded by the built-in algorithm of the analysis software. The sleep variables included total sleep time, sleep efficiency, proportion of time spent in each sleep stage (REM, N1, N2, and N3), sleep latency, wake after sleep onset, awakening index, and microarousal index.

### 2.9. Subjective Outcomes

Subjective sleep quality and disturbances over the past month were assessed using the PSQI-J [41], which consists of seven components, each scored from 0 to 3, with a total score ranging from 0 to 21. Higher scores indicate poorer sleep quality. The PSQI-J was administered three times: before the baseline measurement period and after each intervention phase. For the latter two assessments, participants were explicitly instructed to respond based solely on the preceding intervention phase, ensuring that their recall was confined to the relevant 33-night period.

Additionally, participants rated their morning symptom severity and splint comfort using an 11-point numerical rating scale (NRS) combined with a face rating scale. The questionnaire included the following items: (1) current morning symptom severity (0 = most severe, 10 = no symptoms), (2) improvement in morning symptoms compared to pre-enrollment (0 = much worse, 10 = greatly improved), and (3) splint comfort over the past month (0 = extremely uncomfortable, 10 = extremely comfortable). This questionnaire was administered twice after each splint measurement period. Although the NRS has not been specifically validated for SB or splint trials, it is a widely used and versatile tool for the subjective evaluation of pain and discomfort and is commonly employed in clinical research. In the present study, evaluating participants’ perceived comfort and clinical acceptability of the splints was considered important; therefore, a simple and easily interpretable NRS was selected for these assessments.

### 2.10. Statistical Analysis

For both the EMG parameters and sleep variables, the mean values from the five recording nights for each participant were calculated, and the median values across all participants were used as representative values. Data were excluded from the analysis if participants completed fewer than three of the five nights during each measurement period.

Statistical analyses were performed using IBM SPSS Statistics, version 31 (IBM Corp., Armonk, NY, USA). All analyses were conducted on a per-protocol basis. Only participants who completed all required measurements in both intervention phases were included in the dataset. Comparisons between BBO and CG splints were performed using the Wilcoxon signed-rank test (α = 0.05). As this study was exploratory in nature, no formal adjustment for multiple comparisons was performed; *p*-values are therefore interpreted descriptively.

Because the primary objective of this study was to compare the two guidance schemes, baseline values were intentionally not included in the analysis, and the focus was placed on the intervention phases between BBO and CG. A simple two-condition comparison was performed to evaluate the effects of the BBO and CG splints on the primary and secondary outcomes. Period, sequence, and carry-over effects were not explicitly modeled, which is considered reasonable given the sufficient washout period, immediate and short-term nature of the primary outcome (integrated EMG per hour), and relatively homogeneous study population. Nevertheless, subtle period or sequence effects could not be fully excluded.

## 3. Results

### 3.1. Participants Flow and Baseline Characteristics

Of the 24 enrolled participants (16 men and 8 women; mean age, 26.1 years), 23 participants (12 in the BBO-first sequence and 11 in the CG-first sequence; 15 men and 8 women; mean age, 26.0 ± 2.2 years) completed the study. One participant dropped out of the study because of sleep disturbances caused by the recording devices. Analyses included on the remaining 23 participants who completed all required measurements in both intervention phases (per-protocol analysis).

All participants completed the daily splint-use logs. The average reported splint-wearing compliance in the BBO-first sequence group was 97% and 99% during the first and second intervention phases, respectively, and 100% and 99% in the CG-first sequence group, indicating that generally high adherence to the prescribed usage schedule.

Regarding the 23 participants, the mean number of residual teeth was 27.9. The natural occlusal guidance patterns of the participants were as follows: 10, 8, 3, 1, and 1 participants had bilateral CG, bilateral group function, CG on one side and group function on the other, group function on one side and first molar guidance on the other, and bilateral balancing-side guidance, respectively.

### 3.2. EMG Parameters

Exclusions due to poor signal quality or invalid recordings are summarized as follows. In the BBO-first sequence group (*n* = 12), 11 and 7 nights were excluded during the first (5 nights × 12) and second intervention phases (5 nights × 12), respectively, corresponding to mean percentages of 10% and 3% based on individual exclusion rates. In the CG-first sequence group (*n* = 11), 5 and 6 nights were excluded during the first (5 nights × 11) and second intervention phases (5 nights × 11), respectively, corresponding to mean percentages of 9% and 11% based on individual exclusion rates. A chi-square test indicated no significant difference in the number of excluded nights between groups (*p* = 0.41). However, all available EMG recordings from every participant met the predefined data inclusion criteria throughout all study phases and were included in the analysis. Overall signal quality was consistent between the BBO and CG sessions.

No statistically significant differences were found for any of the EMG parameters (Table 1). The effect sizes for integrated EMG per hour, number of episodes per hour, number of bursts per hour, mean episode duration, and total episode duration per hour were 0.18, 0.18, 0.10, 0.11, and 0.20, respectively, indicating small effects [42]. These values were consistently lower for the BBO splint compared with the CG splint, although the differences were small and should be interpreted cautiously given the small sample size. Additionally, both splints exhibited consistently lower values across all EMG parameters than at baseline.

**Figure 5:**
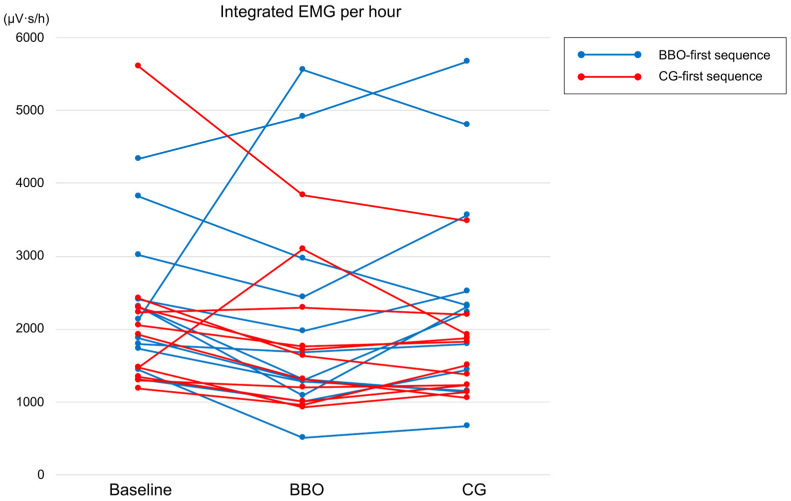
Individual integrated EMG per hour (μV·s/h) values during sleep for each participant (*n* = 23) under baseline, BBO, and CG conditions. Each line represents data from a single participant across the three conditions. BBO, bilateral balanced occlusion; CG, canine guidance.

Figure 5 shows individual integrated EMG per hour (μV·s/h) values, the primary outcome, for each participant (*n* = 23), illustrating individual within-subject changes across the baseline, BBO, and CG conditions.

**Figure 6:**
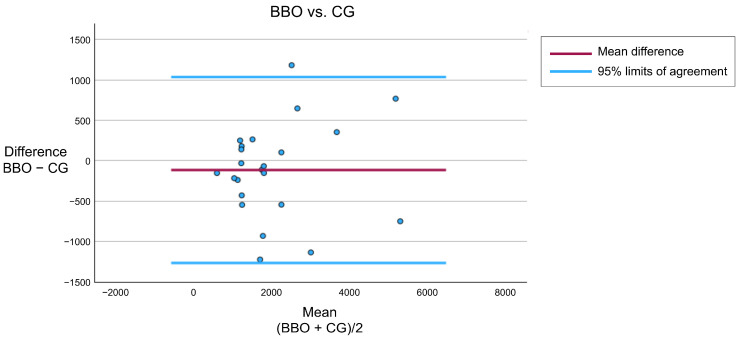
Bland–Altman plots for integrated EMG per hour (μV·s/h). The plots depict the differences between BBO and CG conditions for each participant (*n* = 23) against their mean values. The red line represents mean difference, and blue lines indicate 95% limits of agreement (mean difference ± 1.96 × standard deviation), illustrating within-subject variability in this crossover study.

Bland–Altman plots for integrated EMG per hour (μV·s/h) values are presented in Figure 6, depicting the distribution of differences and limits of agreement, thereby providing a visual assessment of within-subject variability in this crossover study. The mean difference in integrated EMG per hour (μV·s/h) was close to zero, indicating no systematic bias between the two conditions. Most data points fell within the 95% limits of agreement, suggesting that the variability of within-subject differences was largely random rather than condition-dependent. Although some dispersion was observed, consistent with the substantial inter-individual variability often reported in SB, there was no apparent trend indicating proportional bias.

### 3.3. Sleep Variables

All PSG recordings from every participant met the predefined data inclusion criteria throughout all study phases and were included in the analysis. Overall data quality was consistent between the BBO and CG sessions.

No significant differences were observed between the BBO and CG groups for any sleep variable (Table 2). Effect sizes were generally small (*r* = <0.01–0.19), with the largest observed for Stage N1 (%) (*r* = 0.31, *p* = 0.215), awakening index (/h) (*r* = 0.18, *p* = 0.482), and microarousal index (/h) (*r* = 0.19, *p* = 0.423), suggesting minimal to modest differences between conditions.

### 3.4. Subjective Outcome Measures

No significant differences were found between the BBO and CG groups in subjective outcomes, including PSQI-J score, morning symptom severity, and splint comfort (Table 3). Effect sizes for all comparisons were small (*r* = 0.01–0.10), indicating minimal practical differences between groups.

## 4. Discussion

This study compared the effects of splints using two different guidance schemes, CG and BBO, on the masseter EMG activity during sleep, sleep architecture, and subjective outcomes in adults with SB. There were no statistically significant differences between the two guidance schemes for any of the evaluated variables. Given the small sample size and variability inherent to SB, these non-significant findings should not be interpreted as demonstrating equivalence between the splint types. Instead, they suggest that any short-term differences, if present, were likely small and may require a larger sample to detect. Accordingly, the influence of strategically applied occlusal guidance on SB-related masticatory muscle activity and sleep quality appears limited within the detectable range of this study, although definitive inferences should be made with caution. Importantly, this study moves beyond conventional short-term or simulated-function research conducted during wakefulness, contributing to the advancement of the field. By assessing nocturnal EMG responses in adults with SB following a 4-week splint adaptation period, the study provides physiologically relevant data on masseter activity under two different splint guidance schemes during actual sleep. Clinically, this approach offers more meaningful insights into the impact of guidance differences on nocturnal muscle activity and sleep quality, and may help inform individualized management strategies for SB.

### 4.1. Effect of Occlusal Guidance Schemes on Masticatory EMG Activity

Manns et al. [22] compared the masticatory EMG activity in six healthy individuals wearing two types of splints, one with CG and the other with group function, during simulated mandibular movements while awake. The integrated EMG of the masseter and temporalis muscles was significantly lower with the CG on both the working and non-working sides, leading to the recommendation of the CG for splint design. Conversely, Borromeo et al. [24] compared mean masseter EMG amplitudes during simulated mandibular movements in 10 healthy individuals using a maxillary splint with CG and a mandibular splint with a group function. They found no significant difference between the two guidance types and concluded that splint use itself reduced EMG activity regardless of the guidance design, without indicating the superiority of either guidance type.

These findings alone are insufficient to determine the type of guidance to apply during occlusal splint therapy in patients with SB for several reasons. First, previous studies evaluated masticatory EMG activity during wakefulness, when participants voluntarily controlled the force exerted during grinding or lateral clenching. Particularly in CG, a sense of mandibular instability or fear of tooth fracture may have subconsciously suppressed muscle activity, leading to bias. Contrastingly, such feedback mechanisms are unlikely during sleep, as evidenced by frequent clinical findings of root fractures and severe tooth wear. Indeed, masticatory EMG activity during sleep varies substantially among individuals and can exceed 100% MVC in the intercuspal position (mean 53.1%, range 17.3–111.6%) [43]. Second, mandibular movements during SB are highly variable and often extend beyond the edge-to-edge position of the canines [11,44], challenging the accurate replication of such diverse mandibular movement patterns through standardized simulations during wakefulness. Third, as previous studies were conducted in healthy individuals rather than patients with SB, the external validity and generalizability of their findings remain limited.

In a sleep study, Rugh et al. [25] compared the effects of splints with CG and molar guidance in individuals with SB over 10–14 consecutive nights; no significant differences in the total integrated EMG between the two guidance types were found, consistent with the results of the present study. Nonetheless, their small sample and the evaluation immediately after splint insertion, when SB activity is often transiently suppressed, limit the interpretability of their findings. Because the suppressive effect of splints is time-dependent and tends to diminish as physiological adaptation progresses [9,10], assessments conducted after sufficient adaptation are likely more appropriate for evaluating guidance-related differences, if any.

To date, few studies have examined the longer-term effects of different guidance schemes (e.g., CG, BBO, and group function) while explicitly considering the adaptation process, so evidence remains sparse. The current randomized crossover study contributes to this limited body of work by evaluating guidance schemes after participants had adapted to each splint, reflecting real-world conditions in which patients typically use splints over extended periods. Nonetheless, given the modest sample size and lack of formal assessment of statistical power for detecting smaller effects, the present findings should be interpreted cautiously.

No significant differences were observed in the EMG parameters during sleep between BBO and CG splints, corroborating previous reports indicating that peripheral sensory factors, including occlusal contact, are secondary in the occurrence of SB. Conversely, central and/or autonomic nervous system mechanisms are primarily involved in its genesis [30]. Although current evidence supports this centrally driven model, we initially considered that modifying occlusal contact patterns might still produce subtle differences in peripheral sensory input and, consequently, motor output during sleep. Based on this theoretical assumption, the BBO design was expected to exert at least a minimal biomechanical influence compared with a conventional occlusal splint. The present findings, however, align with the growing body of literature suggesting that such peripheral modifications have limited impact on SB activity, thereby making the logical link between our original hypothesis and the observed null findings more explicit and consistent with current understanding of SB pathophysiology. A previous crossover trial by Harada et al. [9] reported no significant difference in the suppressive effect on SB-related muscle activity between a maxillary stabilization splint and a palatal splint without occlusal coverage after 6 weeks of use, which is consistent with the present results. Nevertheless, the small sample size and absence of formal modeling for smaller effects warrant cautious interpretation of the present findings. Future studies with larger cohorts and more robust analytic approaches are needed to determine whether subtle guidance-dependent differences exist.

### 4.2. Effect on Sleep Architecture and Subjective Outcomes

Analysis using portable PSG revealed no significant differences between BBO and CG in either sleep variables or subjective outcomes. A recent systematic review investigating the effects of CG on splints in individuals with TMD and SB reported that there is insufficient evidence to conclude that any specific occlusal guidance scheme is superior in improving subjective or objective outcomes [45], which is generally consistent with the present findings. Nonetheless, to date, no study has directly compared the effects of these two distinct guidance schemes.

### 4.3. Clinical Implication

The participants in this study were limited to students or dentists affiliated with a university dental hospital and thus may not fully represent the broader population of clinical patients with SB. However, the participants were selected based on diagnostic criteria commonly used in clinical practice, including EMG-based SB episode counts and clinical signs and symptoms assessed through examination and self-report. This group exhibited characteristics frequently encountered among general clinical patients with SB. Therefore, the participants are not considered substantially different from typical patients with SB, and the generalizability of the study findings is likely minimally affected.

This study indicates that incorporating CG into a splint does not reduce masseter EMG activity compared with BBO, with similar levels of muscle activity observed between the two guidance schemes. As this study did not include direct assessments of occlusal force distribution or temporomandibular joint (TMJ) biomechanics, no conclusions regarding mechanical loading can be drawn from the present data. Nevertheless, the biomechanical literature suggests that, if muscle activity during SB is comparable between guidance types, grinding forces under CG may tend to concentrate on the canines, whereas BBO may distribute these forces across multiple teeth and both sides of the arch. Furthermore, SB has been reported to increase loading forces on the TMJ and is considered a potential risk factor for TMJ sounds such as clicking [46,47]. Based on prior theoretical models, the presence of non-working-side contacts in BBO could limit the upward displacement of the ipsilateral condyle compared with CG; however, this mechanism was not measured in the present study.

From the clinical perspective of occlusal force distribution and TMJ load control, BBO may provide theoretical biomechanical advantages as a guidance design for occlusal splints. However, these interpretations are extrapolated from the literature rather than supported by direct measurements from the present study, and should therefore be regarded as hypotheses requiring empirical validation.

### 4.4. Limitations and Future Perspectives

This study had some limitations. First, participants were evaluated uniformly without consideration of individual SB subtypes. The effects of occlusal guidance on EMG activity may differ between individuals with predominantly phasic or tonic episodes [48,49]. Furthermore, the eccentric mandibular movement patterns associated with SB vary across individuals [44,46]; thus, subtype-specific evaluations remain necessary. Additionally, the sample consisted mainly of young adults who were students or dentists, representing a convenience-type and relatively narrow population; therefore, the external validity may be limited.

Second, the sample size calculation was based on effect sizes reported for EMG activity under awake conditions in a previous study [24]. As EMG activity during sleep exhibits greater variability and is primarily centrally regulated [28], comparable effect sizes cannot be assumed. Consequently, the present trial may have been underpowered to detect smaller, potentially clinically meaningful differences in nocturnal EMG or sleep architecture.

Third, the study was conducted without participant or clinician blinding, which may introduce bias. Although a washout period was implemented, the crossover design did not include formal modeling or statistical testing for period, sequence, or carry-over effects. Subtle residual or adaptation effects therefore cannot be fully excluded.

Fourth, baseline measurements were not incorporated into the primary between-group comparisons. Although baseline values are reported descriptively, future studies should include direct baseline–intervention comparisons to better differentiate between general splint effects and guidance-specific influences.

Fifth, EMG recordings were collected from a single side selected individually by each participant, and standardized criteria for side selection were not used. As single-channel EMG has inherent limitations [34], side-dependent measurement variability cannot be ruled out. Sleep architecture was also assessed using a portable PSG system, which may be less accurate than laboratory-based audio-video PSG.

Finally, the study did not assess long-term clinical outcomes such as pain, tooth wear, or TMJ sounds, and it was not powered or designed for non-inferiority testing. Future research should employ larger, more diverse cohorts, incorporate long-term follow-up, include formal crossover modeling, and consider non-inferiority trial designs to more definitively characterize the clinical relevance of occlusal guidance schemes in SB management.

## 5. Conclusions

This randomized crossover study found no significant differences in masticatory muscle activity during sleep, sleep architecture, or subjective outcomes between BBO and CG splints in adults with SB following a 4-week adaptation period. Although the results do not indicate large effects of guidance type, subtle differences cannot be excluded. Speculative biomechanical implications, such as effects on occlusal force distribution or TMJ load, were not directly measured and should be interpreted with caution. Additional limitations—including the convenience sample, short-term follow-up, and absence of formal modeling for period or carryover effects—also constrain the generalizability of these findings. Future research with larger, more diverse cohorts, longer follow-up, and comprehensive PSG assessment is warranted to further clarify the clinical impact of occlusal guidance in SB management.

## Figures and Tables

**Table 1:** EMG parameter comparison between BBO and CG splints.

EMG Parameters	Baseline	BBO	CG	*p*-Value	Effect Size (*r*)
Integrated EMG per hour (μV·s/h)	2044 (1473–2362)	1630 (1138–2363)	1823 (1231–2317)	0.394	0.18
Number of episodes per hour (/h)	11.00 (9.00–11.89)	9.07 (7.71–10.51)	9.66 (8.94–11.53)	0.394	0.18
Number of bursts per hour (/h)	67.64 (52.79–94.02)	49.99 (38.66–74.00)	56.55 (41.33–82.30)	0.627	0.10
Mean episode duration (s/episode)	12.43 (10.50–14.19)	11.82 (9.45–14.29)	11.98 (9.67–14.35)	0.605	0.11
Total episode duration per hour (s/h)	129.1 (107.5–153.4)	123.7 (84.8–138.5)	127.6 (90.4–139.8)	0.330	0.20

*n* = 23. Data are presented as medians (first–third quartiles). EMG parameters were compared between BBO and CG splints using Wilcoxon signed-rank test. Effect sizes (*r*) for nonparametric comparisons between BBO and CG splints were calculated using the formula *r* = *Z*/√*n*, where *Z* is the Wilcoxon signed-rank test statistic and *n* is the total number of participants. Effect sizes were interpreted according to Cohen’s criteria as small (*r* = 0.1), medium (*r* = 0.3), and large (*r* = 0.5) [42]. BBO, bilateral balanced occlusion; CG, canine guidance.

**Table 2:** Sleep variable comparison between BBO and CG splints.

Sleep Variables	Baseline	BBO	CG	*p*-Value	Effect Size (*r*)
Total sleep time (h)	5.3 (5.0–5.8)	5.4 (4.7–5.7)	5.3 (5.0–5.7)	0.653	0.09
Sleep efficiency (%)	87.3 (78.5–89.3)	86.8 (83.2–89.6)	86.8 (79.6–89.1)	0.896	0.03
Stage REM (%)	22.2 (16.7–26.4)	24.2 (17.9–25.8)	21.9 (19.2–24.5)	0.601	0.11
Stage N1 (%)	5.1 (4.0–7.0)	5.8 (4.0–8.1)	5.1 (3.8–6.7)	0.139	0.31
Stage N2 (%)	47.7 (37.8–52.0)	47.9 (39.2–56.8)	48.4 (39.8–54.6)	0.983	<0.01
Stage N3 (%)	27.0 (20.5–32.4)	24.2 (16.6–28.1)	23.6 (19.1–32.1)	0.557	0.12
Sleep latency (min)	11.3 (9.2–15.0)	10.7 (8.2–21.4)	9.3 (5.6–20.0)	0.777	0.06
WASO (min)	38.1 (27.2–68.6)	35.0 (28.8–54.0)	34.7 (25.0–51.0)	0.795	0.05
Awakening index (/h)	10.3 (7.1–13.4)	11.4 (7.6–12.2)	9.6 (6.0–12.3)	0.396	0.18
Microarousal index (/h)	1.4 (1.0–1.7)	1.3 (1.0–1.6)	1.3 (0.9–1.6)	0.360	0.19

*n* = 23. Data are presented as medians (first–third quartiles). Sleep variables were compared between BBO and CG splint groups using Wilcoxon signed-rank test. Effect sizes (*r*) for nonparametric comparisons between BBO and CG splints were calculated using the formula *r* = *Z*/√*n*, where *Z* is the Wilcoxon signed-rank test statistic and *n* is the total number of participants. Effect sizes were interpreted according to Cohen’s criteria as small (*r* = 0.1), medium (*r* = 0.3), and large (*r* = 0.5) [42]. BBO, bilateral balanced occlusion; CG, canine guidance; REM, rapid eye movement; WASO, wake after sleep onset.

**Table 3:** Subjective outcome measure comparison between BBO and CG splints.

EMG Parameters	Baseline	BBO	CG	*p*-Value	Effect Size (*r*)
PSQI-J score	3 (2.0–4.5)	4 (2.5–5.5)	4 (3.0–5.5)	0.903	0.03
Current morning symptom severity ^†^	N/A	9 (7.5–9.0)	8 (5.5–10.0)	0.957	0.01
Improvement in morning symptoms compared with pre-enrollment ^†‡^	N/A	6 (5.0–7.5)	5 (5.0–7.0)	0.763	0.06
Splint comfort over the past month ^†^	N/A	8 (7.0–9.0)	8 (6.5–9.0)	0.632	0.10

*n* = 23. Data are presented as medians (first–third quartiles). ^†^, Participants answered the questions using a numerical rating scale (NRS) ranging from 0 to 10. ^‡^, A total of 19 participants who experienced morning symptoms answered the question. Subjective outcomes were compared between BBO and CG using the Wilcoxon signed-rank test. Effect sizes (*r*) for nonparametric comparisons between BBO and CG splints were calculated using the formula *r* = *Z*/√*n*, where *Z* is Wilcoxon signed-rank test statistic and *n* is the total number of participants. Effect sizes were interpreted according to Cohen’s criteria as small (*r* = 0.1), medium (*r* = 0.3), and large (*r* = 0.5) [42]. BBO, bilateral balanced occlusion; CG, canine guidance; PSQI-J, Japanese version of the Pittsburgh Sleep Quality Index; N/A, not applicable.

## Data Availability

The data presented in this study are available upon reasonable request from the corresponding author due to ethical restriction and with approval from the institutional ethics committee.

## Abbreviations

BBO	Bilateral balanced occlusion
CG	Canine guidance
CONSORT	Consolidated Standards of Reporting Trials
EMG	Electromyography
ICC	Intraclass correlation coefficient
MVC	Maximal voluntary contraction
NRS	Numerical rating scale
PSG	Polysomnography
PSQI-J	Japanese version of the Pittsburgh Sleep Quality Index
REM	Rapid eye movement
SB	Sleep bruxism
SPIRIT	Standard Protocol Items: Recommendations for Interventional Trials
TMD	Temporomandibular disorder
TMJ	Temporomandibular joint
WASO	Wake after sleep onset
